# Multimorbidity after USAID

**DOI:** 10.1177/26335565261460403

**Published:** 2026-07-16

**Authors:** Justin Dixon, Efison Dhodho

**Affiliations:** 1 The Health Research Unit Zimbabwe, Biomedical Research and Training Institute, Harare, Zimbabwe; 2 Department of Global Health and Development, London School of Hygiene and Tropical Medicine, London, UK; 3 Organisation for Public Health Interventions and Development, Harare, Zimbabwe

**Keywords:** multimorbidity, global health, integration, localisation, self-reliance, learning health systems

## Abstract

Multimorbidity—the coexistence of multiple chronic conditions within a single individual—has emerged as a powerful lens through which to understand the growing mismatch between population health needs and health systems historically organised around single diseases. In many lower-income countries, multimorbidity has reinvigorated calls for more integrated and person-centred models of care. Yet responses have often been fragmented and incremental, remaining tethered to the vertical programmes and disease-specific architectures they seek to overcome. The recent disruption of USAID funding represents a critical juncture for health systems that have long relied on externally funded programmes. While the immediate consequences for service delivery are substantial, this moment also presents an opportunity to move beyond piecemeal integration towards more fundamental health system transformation. Realising this opportunity will require greater emphasis on locally led adaptation, recognising that multimorbidity is experienced and managed within highly contextual social, organisational and epidemiological realities. Learning health systems offer a promising conceptual horizon for this transition by providing a framework through which health systems can continuously generate, interpret and act upon knowledge in response to changing needs. The challenge ahead is not simply sustaining services, but building more integrated, adaptive and person-centred systems capable of responding to shifting realities of multimorbidity.

Multimorbidity, or the co-occurrence of two or more long-term conditions in one person, affects an estimated 37.2% of adults^1^ and has been described as the next global pandemic. In lower-income countries, the profile of multimorbidity differs from that in higher-income ones^2^ and has been characterised as a formidable syndemic of persisting chronic infectious and rapidly rising non-communicable diseases (NCDs)^2,3^ for which current health systems organised around HIV, TB, and malaria remain vastly unprepared. Calls for integrated, person-centred health systems have been strongly advocated for, but action has remained hamstrung by fragmented, unevenly-resourced vertical programmes for priority diseases.^4^ The USAID and related funding cuts, however, have shaken the foundations of global health and, for all their devastation, present opportunity to elevate multimorbidity within broader efforts to build stronger, more responsive health systems post-USAID.

## Have we been too timid?

Despite the momentum building around multimorbidity^4^ and burgeoning North-South and South-South research collaborations to address its different dimensions, its potential to transform the design and delivery of healthcare in lower-resource health systems remains contested and, as yet, unrealized.^5,6^ Commentators have observed that multimorbidity, although evidencing the need to tackle disease clusters and interactions holistically rather than one-at-a-time, has yet to sufficiently unsettle the vertical, disease-specific models of funding, research, policy and practice that have come to predominate many health systems through the expansion of disease-specific global health initiatives over several decades, undermining earlier progress towards comprehensive primary healthcare and fuelling the fragmentation and uneven resourcing of services.^6^ The rapid influx of external support that arrived on the back of the HIV response in the late 1990s clearly showed that population-level chronic disease control is achievable in resource-limited settings. As HIV programmes have matured over the last 25 years and increasing numbers of people living with HIV experience mutimorbidity as they age, HIV has been an obvious point of departure for many researchers and programmers seeking to better understand this emerging challenge and to test the waters of integrating in other chronic diseases.^7^ Upon the conceptual and material infrastructure built up around HIV, we have seen the development and expansion of differentiated service delivery (DSD) to include NCDs (as well as other concurrent health needs), with earlier-stage initiatives to extend DSD principles further to address NCDs and multimorbidity among the general population, often under the guise of an integrated ‘chronic care’ model.^8^ However, modest progress with integrating NCDs into HIV services^9^ and far greater challenges faced in expanding population-wide integrated chronic care beyond research and programme-funded contexts^6^ suggests that a bolder, more radical break from business-as-usual is needed. As long the primary foothold of multimorbidity within low-resource health systems is in the gradual, incremental expansion of vertical programmes or even their ‘rapid modifications’, it may never adequately extend access to integrated services to all those who need them and may even inadvertently keep certain high-priority diseases at the centre at the expense of whole persons.^6^

## A fork in the road Post-USAID

We have reached a fork in the road with the cessation of USAID funding. Some forewarn that in regions like Sub-Saharan Africa where 55% of foreign assistance came from USAID, shrinking health budgets will result in a doubling down on a vertical, disease-specific approach, undermining gains in integration and NCD care.^10^ We, like others, more optimistically contend that amidst the devastation could be a moment for true change.^11^ Previous sluggishness or reluctance to expand integration remits may be inverted such that the goals of sustainability may now be served by full rather than partial or incremental integration. There will unlikely be resources for self-standing HIV clinics and parallel systems of research, surveillance, service delivery, and monitoring and evaluation (M&E), making integrated chronic care no longer simply a matter of ethical imperative, clinical benefit, or cost-effectiveness but brute resource necessity. Recognising this, some Ministries of Health (e.g. Uganda’s) have issued directives for all self-standing HIV structures to be phased out and integrated into general outpatient services alongside other chronic conditions. Numerous others are considering similarly drastic changes. The tectonics are shifting, and health systems may be hospitable to integration in a way that has previously been impeded by programmatic structures.

However, without strategic, multi-level action to leverage the potential gravity shift, the opportunity will be lost. Decisive action is needed by northern governments, funders, and research institutions, many of whom, particularly from the UK, have actively shaped and promoted the multimorbidity agenda^4^ and can be a leading voice for placing multimorbidity at the forefront of global health reform. Yet, transforming the multimorbidity response means supporting a concurrent sovereignty shift in agenda-setting towards national and sub-national actors.^11^ This is necessary not only in the interests of greater self-reliance and sustainability, but because of the place-specific nature of multimorbidity itself. Multimorbidity has the apparently simple definition of two-or-more long-term conditions. But as the syndemic framework compellingly shows, the way biological, social, and structural factors intersect to favour specific disease clusters and with what impacts on health and health systems is complex, contextual, and dynamic.^3^ How multimorbidity presents in a given setting, what it would mean to ‘phase out’ self-standing HIV structures, and which ‘integrated’, ‘person-centred’, and ‘differentiated’ services should take their place, can vary greatly by national, sub-national, or even facility context. Those best positioned to recognise and respond to local manifestations of multimorbidity are therefore local researchers, decision-makers, frontline carers, and affected communities: those who intimately understand local case mixes, priorities, and expectations for care; the inefficiencies, duplications, and harms of rigid, siloed programming; and the value of older, dormant structures of comprehensive primary care. A shift in the locus of knowledge from external programmes to domestic collectives is crucial for realigning health system priorities with the complex, evolving needs of whole persons.

## Learning to learn: A path forward

Calls for bottom-up responses in global health abound and are often prematurely placed on the shoulders of incapacitated health system leaders. But not only can health systems now ill-afford but to take the reins within the vacated spaces vacated by foreign actors, but there are practical frameworks to support health systems to build requisite capacities ‘in-house’ for greater self-reliance.^12^ The learning health systems (LHS) framework, for example, pinpoints learning as a key pillar of health system strengthening.^12,13^ Collapsing divisions between ‘knowing’ and ‘doing’, within LHS knowledge generation and use are embedded and incentivised within routine service delivery for continuous, bottom-up improvement.^13^ Health systems built on principles of LHS not only provide a stronger, more sustainable foundations for selecting and adapting evidenced-based interventions, but promote organic, bottom-up change by decentralising knowledge and innovation towards the frontline. While relevant across the spectrum of health, learning infrastructure of salience for building more multimorbidity-responsive services and systems post-USAID includes integrated, accessible health information systems monitoring whole health rather than single diseases (e.g., via electronic health records); embedded research and knowledge translation platforms for inter-condition collaboration and co-production; and pilot or learning sites from which to advance and diffuse models of integration and differentiation.^14^

Multimorbidity is notoriously challenging to define, quantify, and translate into evidence-based interventions. But while it continues to be an important focus of academic global health, *enough* is known to continue to build the knowledge base from a position of ‘doing’, empowering those best placed with confidence and capacities to do so. The imperative to ground new interventions in ‘universal’ knowledge sits at the core of evidence-based global health but has long favoured northern actors with the resources for high-end research and programme infrastructures – which, for all their gains, are responsible for the rigid, siloed systems that have enabled multimorbidity to hide in plain sight.^15^ With the dominant knowledge architectures of global health at breaking point, and with neither the time nor the resources for protracted evidence-to-action pathways, there is unprecedented need to forge more dynamic, bi-directional relations between knowledge, policy and practice to make multimorbidity visible and actionable within post-USAID health systems. ‘Learning to learn’ is a steep curve, to be sure.^13,14^ But this is paramount if we are to harness the shifting gravity of the current moment to support health systems to confront rapidly evolving multimorbidity syndemics with increasingly threadbare external funding and support.
